# Tooth Loss in the United Kingdom – Trends in Social Inequalities: An Age-Period-and-Cohort Analysis

**DOI:** 10.1371/journal.pone.0104808

**Published:** 2014-08-08

**Authors:** Eduardo Bernabé, Aubrey Sheiham

**Affiliations:** 1 King’s College London Dental Institute at Guy’s, King’s College and St. Thomas’ Hospitals, Division of Population and Patient Health, London, United Kingdom; 2 Department of Epidemiology and Public Health, University College London, London, United Kingdom; UNC School of Dentistry, University of North Carolina-Chapel Hill, United States of America

## Abstract

This study assessed trends in social inequalities in tooth loss in the United Kingdom between 1988 and 2009. Data from 20,126 adults who participated in the latest three national Adult Dental Health Surveys in England, Wales and Northern Ireland were used. Social class was determined using the 6-point Registrar General’s Social Class. Three indicators of tooth loss were analysed; the proportion of edentate people among all adults and the number of teeth and the proportion with functional dentition (defined as having 20+ teeth) among dentate adults. Trends were modelled within an age, period and cohort framework using partial least squares regression (PLSR). Confidence intervals for PLSR estimates were obtained using non-parametric bootstrapping. The Slope and Relative Index of Inequality (SII and RII) were used to quantify social inequalities in tooth loss. Between 1988 and 2009, absolute inequalities in total tooth loss narrowed (SII changed from −28.4% to −15.3%) while relative inequalities widened (RII from 6.21 to 20.9) in the whole population. On the other hand, absolute and relative social inequality in tooth loss remained fairly stable over time among dentate adults. There was an absolute difference of 2.5–2.9 in number of teeth and 22–26% in the proportion with functional dentition between the lowest and highest social classes. In relative terms, the highest social class had 10–11% more teeth and 25–28% higher probability of having functional dentition than the lowest social class. The findings show pervasive inequalities in tooth loss by social class among British adults despite marked improvements in tooth retention in recent years and generations. In the whole adult population, absolute inequalities in tooth loss have narrowed while relative inequalities have increased steadily. Among dentate adults, absolute and relative inequalities in number of teeth and proportion of people with functional dentition have remained significant but unchanged over time.

## Introduction

Tooth loss is an outcome that reflects not only the individuals’ history of dental diseases but also patients’ and dentists’ attitudes and behaviours, the availability and accessibility of dental services and the prevailing philosophies of dental care [1], [2]. It is considered an effective marker of population oral health and is therefore monitored in many countries. Despite large declines in the prevalence and incidence of tooth loss at global, regional and country levels in the past two decades [3], socioeconomic inequalities in tooth loss persist even in developed countries [4]–[8]. There is a social gradient in tooth loss, regardless of how the latter is measured, either as the prevalence of total tooth loss, the number of teeth or the proportion of adults with functional dentition, commonly assessed as having 20 or more natural teeth.

Monitoring social inequalities in health over time is important to improve understanding of the social determinants of health and evaluate policies to promote health and reduce health inequalities [9], [10]. Some recent studies have explored the trends in social inequality in tooth loss [8], [11]–[14]. Cunha-Cruz et al. [11] showed that for 25–74 year-old Americans, absolute differences in total tooth loss between high and low socioeconomic groups remained stable from 1972 to 2001. Dye and Thornton-Evans [12] also reported little change in the absolute difference of no tooth loss between poor and non-poor groups from 1988/94 to 1999/04 for 35–44 year-old Americans, but the absolute difference in total tooth loss between poor and non-poor groups increased from 19% to 23% for 65–74 year-olds. Elani et al. [8] showed that for adults aged 20 years and older, absolute differences in total tooth loss by income remained wider in Canada (7.5% in 1970/72 to 4.0% in 2007/09) compared to the US (3.7% in 1971/74 to 3.1% in 2007/08), while absolute differences by education narrowed (13.8% to 2.8% in Canada and 9.0% to 3.6% in the US). Celeste et al. [14] found that the absolute difference in total tooth loss between the poorer and the richer groups decreased in Brazil from 1986 to 2002, and in Sweden, from 1968 to 2000, while relative differences remained the same (prevalence ratios of 2.58 and 2.70 for Sweden and 1.67 and 1.12 for Brazil, with no significant trends over time). Holst [13] reported that the absolute difference in total tooth loss between the highest and lowest income quintiles decreased by 10.5% from 1975 to 2002 in Norwegian adults, with changes of −0.1%, 9.8% and 5.4% for 20–34-, 35–59- and 60+ year-olds respectively, whereas relatively inequality increased from a prevalence ratio of 2.1 in 1975 to 14.2 in 2002, with decreases from 3.5 to 0 in 20–34 year-olds and 2.1 to 1.0 in 35–59- year-olds but an increase from 2.0 to 7.5 in 60+ year-olds. However, the absolute difference in the proportion of people with functional dentition between the highest and lowest income quintiles increased by 25% from 1985 to 2002, with increases of 3.9% and 10.3% in the two youngest groups but a decrease of 9.3% in the oldest group, whereas relative inequality in functional dentition remained stable; from 0.5 in 1985 to 0.9 in 2002, with even smaller changes in the three age groups [13].

All the aforementioned studies used the range, either absolute or relative, to describe inequalities in tooth loss over time, in spite of the fact that such measures overlook changes in the intermediate socioeconomic groups, do not take into account the sizes of the groups being compared and tell little about how these inequalities are affected by the distribution of disease [15], [16]. Alternative measures of health inequalities are thus recommended [17], [18]. More importantly, all previous studies assume that observed changes over survey years are due to period effects, after accounting for age effects. Since age, period and cohort effects are linearly dependent (cohort = period-age), they implicitly assume that cohort effects do not exist. Age, period and cohort effects refer to some type of time-related variation in the outcome of interest, but they have distinct meanings [19], [20]. Age effects refer to variation associated with different age groups. Thus, age effects reflect the biological and social processes of aging internal to individuals and represent developmental changes across the life course. Period effects refer to variation over time periods or calendar years that affect all age groups simultaneously. Lastly, cohort effects refer to variation among groups born in different years [19]. Thus, not taking into account cohort effects may mask trends of social inequalities in health [21]–[23].

As large inequalities in oral health in adults have been reported in the United Kingdom (UK) [7], [24] and improvements in oral health have occurred in children [25] and adults [26], we hypothesised that the pervasive social inequalities in tooth loss in adults have persisted, despite marked improvements in oral health in recent surveys and generations. Within an age, period and cohort framework, this study assessed the trend of social inequalities in tooth loss in the UK between 1988 and 2009.

## Materials and Methods

### Data source

The Adult Dental Health Survey (ADHS) is a national cross-sectional survey in the UK, first carried out in 1968 and repeated each decade thereafter. We used individual-level data from the latest three surveys (1988, 1998 and 2009) in England, Wales and Northern Ireland because Scotland did not participate in 2009 and data from earlier surveys are only available at aggregate level. Each survey is based on a nationally representative sample of adults aged 16 years and over, with participants selected using stratified multi-stage random sampling methods. The survey has two main components; an interview followed by a clinical examination for dentate adults only. Participation rates in the ADHS have varied between 74% and 94% for the interview and between 61% and 82% for the clinical examination, across surveys [27]–[29].

We used two analytical samples in this study (Table 1). The first included 20,126 adults who completed the interview and had no missing data in the variables selected for analysis, whereas the second included 12,206 dentate adults who were clinically examined and had no missing data (93% and 94% of the total number of adults and dentate adults that participated in the three surveys in England, Wales and Northern Ireland, respectively).

**Table 1:** Participation rates in the Adult Dental Health Surveys in England, Wales and Northern Ireland, by survey year.

Unweighted sample	1988	1998	2009	All
*Eligible adults*	5670	5560	13509	24739
No interview obtained	427	560	2129	3116
*Interviewed adults*	5243	5000	11380	21623
Edentate adults	1108	672	813	2593
Dentate adults	4135	4328	10567	19030
*Examined adults with teeth*	3405	3149	6469	13023
*Analytical sample*				
Adults with complete data	5048	4770	10308	20126
% of interviewed adults	96%	95%	91%	93%
Dentate adults with complete data	3307	2963	5936	12206
% of examined adults	97%	94%	92%	94%

### Socioeconomic classification

The 1988 and 1998 ADHS measured social class on the basis of occupation of the household reference person (HRP), formerly Registrar General’s Social Class (RGSC), and grouped individuals with similar levels of occupational skill using an ordinal scale with six categories: professional (I); managerial and technical (II); skilled non-manual (IIINM); skilled manual (IIIM); partly skilled (IV); and unskilled (V). For RGSC, the HRP is defined as the head of household: the oldest householder, with men taking precedence over women in the case of couples or non-related joint householders. In 2009, the ADHS adopted the National Statistics Socioeconomic Classification (NS-SEC) which is derived from HRP’s occupational unit group, employment status and size of establishment, and includes 14 operational categories that can be collapsed into eight analytical classes: higher managerial and professional (1), lower managerial and professional (2), intermediate (3), small employers and own account workers (4), lower supervisory and technical (5), semi-routine (6), routine (7) and never worked and long-term unemployed (8). For NS-SEC, the HRP is defined as the person responsible for owning or renting or who is otherwise responsible for the accommodation. In the case of joint householders, the person with the highest income takes precedence and becomes the HRP. Where incomes are equal, the oldest person is taken as the HRP. The operational categories of NS-SEC can be aggregated to produce an approximated version of RGSC and these approximations have been shown to achieve an overall continuity level of 87% [30].

### Outcome measures

During interviews, participants were asked whether they still have some natural teeth or have lost them all. Dentate participants who completed the interview were invited to a subsequent clinical examination where the condition of all teeth, including third molars, was recorded [27]–[29]. Three indicators of tooth loss were selected for this study. They were the proportion of edentate people among all adults; second, the number of teeth and third, the proportion of people with functional dentition (i.e. defined as having 20 or more teeth) among dentate adults.

### Covariates

During interviews, participants provided information on country of residence, sex and age. Age was grouped into eight 10-year brackets (16–24, 25–34, 35–44, 45–54, 55–64, 65–74 and 75+ years). Period was defined as survey year and coded as 0 for 1988, 1 for 1998 and 2 for 2009. Individuals were grouped into eight 10-year cohort bands by their year of birth. These cohorts were defined by their mid-point; for example, the cohort 1948 included individuals born between 1944 and 1953. The eight cohort groups were 1908, 1918, 1928, 1938, 1948, 1958, 1968 and 1978.

### Measures of social inequalities in tooth loss

We used the Slope Index of Inequality (SII) and the Relative Index of Inequality (RII) to measure, respectively, absolute and relative social inequality in tooth loss in 1988, 1998 and 2009. The SII is the linear regression coefficient that shows the relation between the level of health (i.e. number of teeth) or the frequency of a health problem (i.e. proportion of edentate people or having 20+ teeth) in each socioeconomic category and the hierarchical ranking of each socioeconomic category on the social scale [16]. SII can be interpreted as the absolute change in health level or in the frequency of a health problem when moving from the lowest through the highest socioeconomic level [15], [17]. Two versions of the RII are often used in the literature. The RII for the mean or RII_(mean)_ is calculated dividing the SII by the mean level of population health or by the frequency of the health problem in the population whereas RII for the ratio or RII_(ratio)_ is calculated diving the regression model’s predicted value at the highest point by the regression model’s predicted value at the lowest point [16], [18]. Confidence intervals for SII and RII were obtained as described elsewhere [18], [31].

### Statistical analysis

Each outcome was modelled separately, including social class, sex, age groups, country of residence, period and cohort as explanatory variables. As age, period and cohort are mathematically related (period-age = cohort), they cannot be entered into the same regression model due to perfect co-linearity [19], [32]. To address this problem, we used partial least squares regression (PLSR) to separate and account for age, period and cohort effects [33]. PLSR does not use the original collinear covariates in the estimation process but extracts weighted components that maximise the covariance between the outcome and successively extracted components. The outcome is then regressed onto these components, and corresponding regression coefficients are calculated using linear algebra [34]. The first few components usually explain most of the covariance with the outcome. Hence, increments in the explained variation in the outcome (R^2^) were used as a criterion for selecting the number of PLSR components. Small changes in R^2^ resulting from the inclusion of an additional component indicated the model without such component was preferred [33], [34].

PLSR modelling was undertaken using R-3.1.0 for Windows. We modelled the proportion of edentate people and those having 20+ teeth, using logistic PLSR and number of teeth using linear PLSR. To improve stability in the iteration process for PLSR, all explanatory variables were centred at the mid-point of their respective scales and each converted to a set of indicator (dummy) variables before analysis [33]. As there is no distribution assumption for PLSR coefficients, 1000 non-parametric bootstraps were drawn to obtain 95% confidence intervals (CI) for estimates from PLSR. All analyses were weighted to take account of the survey design and possible non-response bias.

For each outcome, we first present PLSR estimates (adjusted odds ratios and regression coefficients for dichotomous and continuous outcomes respectively), followed by stratified results by survey year (period) to inspect for trends in social inequalities in tooth loss. In the stratified analysis by period, predicted probabilities for dichotomous outcomes and predicted means for continuous outcomes were reported along with the corresponding SII, RII_(mean)_ and RII_(ratio)_.

## Results

The socio-demographic composition of the two analytical samples is shown, by periods, in Table 2. The proportion of edentate adults decreased over the last two decades, from 20% (95% CI: 19–21%) in 1988 to 12% (11–13%) in 1998 to 6% (5–6%) in 2009. These changes were accompanied by increases in the mean number of teeth among dentate adults, from 24.3 (24.0–24.5) in 1988 to 24.8 (24.6–25.0) in 1998 to 25.5 (25.4–25.7) in 2009, as well as in the proportion of dentate adults with 20+ teeth, from 83% (81–84%) in 1988 to 85% (84–86%) in 1998 to 87% (86–88%) in 2009.

**Table 2:** Characteristics of the analytical samples, by survey year.

Explanatory	All adults			Dentate adults only		
variables	1988	1998	2009	1988	1998	2009
*Sample size* a	5048	4770	10308	3307	2963	5936
*Sex*						
Male	49%	51%	49%	52%	53%	49%
Female	51%	49%	51%	48%	47%	51%
*Age groups*						
16–24 years	18%	13%	11%	23%	15%	11%
25–34 years	18%	19%	17%	23%	22%	17%
35–44 years	18%	18%	20%	21%	20%	21%
45–54 years	14%	17%	18%	14%	18%	18%
55–64 years	14%	13%	16%	11%	12%	16%
65–74 years	11%	11%	11%	6%	9%	10%
75+ years	8%	9%	9%	2%	4%	7%
*Country*						
England	92%	92%	92%	92%	92%	92%
Wales	5%	6%	5%	6%	5%	5%
Ireland	3%	3%	3%	3%	3%	3%
*Social class* b						
I (highest)	8%	7%	5%	10%	8%	6%
II	27%	29%	30%	31%	31%	31%
IIINM	13%	14%	22%	13%	14%	23%
IIIM	33%	30%	22%	31%	30%	21%
IV	14%	15%	16%	12%	14%	15%
V (lowest)	5%	5%	5%	4%	4%	4%

a Counts are unweighted.

b Social class groups are professional (I); managerial and technical (II); skilled non-manual (IIINM); skilled manual (IIIM); partly skilled (IV); and unskilled (V).

Table 3 shows the results of the PLSR carried out with each outcome. The 95% CI revealed that all estimates (for the comparison of code 1 to code 0 used with each dummy variable) were statistically significant at the 5% level. Marked improvements in tooth retention were observed by periods and cohorts, independent of the negative effect of aging. Social gradients were found in the three outcomes after accounting for country of residence, sex and age, period and cohort effects. In the full sample of adults, the odds of being edentate increased gradually from the highest (I-NM) to the lowest (V-M) social class. Similarly, the number of teeth and odds of having 20+ teeth among dentate adults increased progressively from the lowest to the highest social class.

**Table 3:** Association of social class with three indicators of tooth loss from partial least squares regression (PLSR) for 20126 adults and 12206 dentate adults in the UK.

Explanatoryvariables	All adults		Dentate adults only			
	Edentulous		Number of teeth		Having 20+ teeth	
	OR^a^	(95% CI)	Coef.^b^	(95% CI)	OR^a^	(95% CI)
*Social class* c						
I (highest)	0.27	(0.20, 0.37)	1.53	(1.37, 1.68)	2.61	(2.54, 2.69)
II	0.46	(0.42, 0.51)	0.64	(0.57, 0.70)	1.46	(1.44, 1.47)
IIINM	0.71	(0.69, 0.73)	0.22	(0.18, 0.27)	1.17	(1.16, 1.17)
IIIM	1.43	(1.35, 1.50)	−0.58	(−0.63, −0.53)	0.78	(0.77, 0.78)
IV	1.71	(1.62, 1.81)	−0.79	(−0.89, −0.70)	0.61	(0.6, 0.62)
V (lowest)	2.37	(2.10, 2.67)	−1.78	(−1.97, −1.58)	0.37	(0.36, 0.39)
*Sex*						
Men	0.88	(0.87, 0.89)	0.16	(0.12, 0.19)	0.99	(0.99, 1.00)
Women	1.14	(1.13, 1.15)	−0.16	(−0.19, −0.12)	1.01	(1.00, 1.01)
*Age groups*						
16–24 years	0.00	(0.00, 0.00)	1.83	(1.76, 1.90)	14.54	(14.17, 14.91)
25–34 years	0.17	(0.16, 0.19)	1.95	(1.84, 2.05)	3.78	(3.74, 3.83)
35–44 years	0.25	(0.24, 0.27)	0.90	(0.84, 0.96)	1.77	(1.75, 1.78)
45–54 years	0.76	(0.75, 0.77)	−0.30	(−0.4, −0.19)	1.08	(1.07, 1.08)
55–64 years	1.25	(1.04, 1.50)	−2.33	(−2.49, −2.17)	0.54	(0.54, 0.54)
65–74 years	1.91	(1.86, 1.95)	−2.85	(−3.00, −2.70)	0.46	(0.45, 0.46)
75+ years	3.20	(3.06, 3.34)	−4.67	(−4.93, −4.41)	0.28	(0.27, 0.28)
*Country*						
England	0.89	(0.89, 0.90)	0.56	(0.53, 0.59)	1.31	(1.3, 1.33)
Wales	1.17	(1.16, 1.18)	−0.40	(−0.45, −0.35)	0.86	(0.85, 0.86)
Northern Ireland	1.03	(1.03, 1.03)	−0.80	(−0.89, −0.70)	0.69	(0.68, 0.7)
*Period*						
1988	1.65	(1.62, 1.68)	−0.42	(−0.44, −0.41)	0.82	(0.82, 0.83)
1998	1.09	(1.09, 1.10)	−0.05	(−0.09, −0.01)	1.04	(1.03, 1.04)
2009	0.59	(0.58, 0.60)	0.46	(0.43, 0.49)	1.14	(1.13, 1.15)
*Cohort*						
1908	7.20	(6.70, 7.75)	−7.01	(−7.30, −6.71)	0.17	(0.16, 0.17)
1918	3.20	(3.06, 3.35)	−5.67	(−5.96, −5.38)	0.19	(0.19, 0.19)
1928	2.27	(2.20, 2.35)	−3.45	(−3.57, −3.33)	0.35	(0.35, 0.36)
1938	1.35	(1.33, 1.37)	−1.80	(−1.86, −1.75)	0.54	(0.54, 0.55)
1948	0.68	(0.67, 0.69)	0.01	(−0.10, 0.12)	0.92	(0.91, 0.92)
1958	0.24	(0.23, 0.25)	1.11	(1.05, 1.17)	1.88	(1.86, 1.89)
1968	0.23	(0.22, 0.25)	1.62	(1.57, 1.67)	3.44	(3.39, 3.49)
1978	0.06	(0.05, 0.07)	1.55	(1.45, 1.65)	10.19	(9.99, 10.39)
1988	0.01	(0.00, 0.06)	2.24	(2.12, 2.35)	8.35	(7.99, 8.73)

a Odds ratios (OR) from logistic PLSR.

b Regression coefficients from linear PLSR.

c Social class groups are professional (I); managerial and technical (II); skilled non-manual (IIINM); skilled manual (IIIM); partly skilled (IV); and unskilled (V).

Table 4 reports the predicted probability of being edentate for all adults as well as the predicted number of teeth and probability of having 20+ teeth (with 95% CI) for dentate adults, by social class and period, calculated from their respective PLSR models. Monotonic gradients in the three outcomes were found by social class in every survey despite overall improvements over time. These findings were used to calculate measures of social inequalities in tooth loss, which were thus adjusted for all covariates (Table 5). Among all adults, absolute inequality in total tooth loss diminished over the two decades, as indicated by the change in SII from −28.4 in 1998 to −15.3 in 2009. The SII indicated that the probability of being edentate decreased by 15.3% from the lowest through the highest social class in 2009. The RII_(mean)_ varied from 1.65 in 1988, suggesting that the SII was 1.65 times the overall probability of being edentate, to 1.55 in 2009. And the RII_(ratio)_ increased from 6.21 in 1998, implying that the probability of being edentate in the lowest social class was 6.21 times the probability in the highest social class, to 20.9 in 2009. On the other hand, measures of social inequality in tooth loss among dentate adults remained fairly stable over time. SII was positive and varied between 2.5 and 2.9 for the predicted number of teeth (indicating that the highest social class had 2.5–2.9 more teeth than the lowest social class) and between 21.8 and 25.8 for the probability of having 20+ teeth. The RII_(mean)_ varied from 0.10 to 0.12 for the predicted number of teeth and from 0.28 to 0.32 for the probability of having 20+ teeth; and the RII_(ratio)_ varied between 0.89 and 0.90 for the predicted number of teeth and between 0.72 and 0.75 for the probability of having 20+ teeth.

**Table 4:** Predicted probabilities of being edentate for adults and predicted number of teeth and probability of having 20+ teeth for dentate adults in the UK, by survey year.

Surveyyear	Socialclass^a^	All adults		Dentate adults only			
		% edentate		Number of teeth		% with 20+ teeth	
		%	(95% CI)	Mean	(95% CI)	%	(95% CI)
1988	I (highest)	7%	(7%, 8%)	25.3	(25.2, 26.2)	89%	(86%, 91%)
	II	11%	(10%, 11%)	24.2	(24.1, 24.1)	80%	(79%, 81%)
	IIINM	17%	(16%, 18%)	23.9	(23.8, 24.1)	77%	(75%, 79%)
	IIIM	25%	(24%, 27%)	23.2	(23.1, 23.3)	73%	(73%, 74%)
	IV	28%	(27%, 30%)	23.2	(23.0, 23.3)	68%	(63%, 72%)
	V (lowest)	34%	(32%, 36%)	22.5	(22.4, 22.6)	62%	(46%, 76%)
1998	I (highest)	4%	(3%, 4%)	25.4	(25.3, 25.5)	89%	(80%, 94%)
	II	6%	(6%, 7%)	25.0	(24.9, 25.0)	87%	(86%, 87%)
	IIINM	11%	(11%, 11%)	24.4	(24.2, 24.6)	84%	(84%, 85%)
	IIIM	20%	(20%, 21%)	23.5	(23.5, 23.6)	75%	(24%, 97%)
	IV	21%	(20%, 22%)	23.1	(23,0, 23.2)	70%	(65%, 75%)
	V (lowest)	26%	(26%, 27%)	22.6	(22.5, 22.8)	64%	(58%, 70%)
2009	I (highest)	1%	(1%, 2%)	26.2	(26.1, 26.3)	95%	(93%, 97%)
	II	4%	(3%, 4%)	25.3	(25.2, 25.5)	87%	(86%, 87%)
	IIINM	5%	(4%, 5%)	24.8	(24.6, 25.0)	84%	(73%, 92%)
	IIIM	11%	(10%, 11%)	24.1	(24.0, 24.2)	78%	(77%, 79%)
	IV	13%	(12%, 14%)	24.1	(23.9, 24.2)	74%	(70%, 77%)
	V (lowest)	18%	(16%, 20%)	22.6	(22.4, 22.8)	55%	(51%, 59%)

Predicted probabilities and predicted means were estimated from logistic and linear PLSR models (adjusted for country of residence, sex and age, period and cohort effects), respectively.

a Social class groups are professional (I); managerial and technical (II); skilled non-manual (IIINM); skilled manual (IIIM); partly skilled (IV); and unskilled (V).

**Table 5:** Health inequality measures (with 95% confidence intervals) for the association of social class with three indicators of tooth loss for 20126 adults and 12206 dentate adults in the UK, by period.

Period	Measure^a^	All adults		Dentate adults only			
		% edentate		Number of teeth		% with 20+ teeth	
1988	SII	−28.4	(−35.0, −21.7)	2.5	(1.4, 3.6)	21.8	(14.4, 29.2)
	RII_(mean)_	1.65	(1.38, 1.92)	0.10	(0.07, 0.14)	0.28	(0.22, 0.35)
	RII_(ratio)_	6.21	(5.07, 7.61)	0.90	(0.87, 0.93)	0.75	(0.70, 0.80)
1998	SII	−24.9	(−33.8, −16.2)	2.9	(2.5, 3.4)	25.5	(16.9, 34.0)
	RII_(mean)_	2.07	(1.55, 2.58)	0.12	(0.11, 0.14)	0.32	(0.24, 0.39)
	RII_(ratio)_	14.89	(10.82, 20.49)	0.89	(0.87, 0.90)	0.72	(0.68, 0.78)
2009	SII	−15.3	(−22.7, −8.0)	2.6	(1.4, 3.8)	25.8	(7.5, 44.1)
	RII_(mean)_	1.55	(1.03, 2.07)	0.10	(0.07, 0.14)	0.32	(0.16, 0.47)
	RII_(ratio)_	20.94	(15.10, 29.05)	0.90	(0.87, 0.93)	0.73	(0.63, 0.83)

a SII: Slope Index of Inequality; RII_(mean)_: Relative Index of Inequality for the mean; and RII_(ratio)_: Relative Index of Inequality for the ratio.

## Discussion

There were pervasive inequalities in tooth loss by social class in the UK adult population. The findings on inequality are consistent with other UK reports on tooth loss [7], [24]. However, we found that social gradients in tooth loss persisted through the two decades studied, regardless of the outcome used (total tooth loss, number of teeth or functional dentition) and despite marked improvements in tooth retention in recent years and generations.

Different trends in social inequality in tooth loss were observed for dentate adults and the whole population. For dentate adults, the SII and RII indicated that social inequalities in number of teeth and proportion with functional dentition have remained significant over the last two decades. There was an absolute difference of 2.5–2.9 in number of teeth and 22–26% in the proportion with functional dentition between the lowest and highest social classes. In relative terms, the top social class had 10–11% more teeth and 25–28% higher probability of having functional dentition than the bottom social class. On the other hand, the SII and RII showed opposite trends for the full population. While the SII implied that absolute inequality in total tooth loss between the highest and lowest social classes decreased over the past two decades (from −28.4% to −15.3%), the RII_(ratio)_ indicated that relative inequality in total tooth loss increased steadily over the same period (from 6 to almost 21 times). Dramatic changes in the epidemiology of total tooth loss could explain the above findings. It has been previously suggested that relative differences may increase while absolute differences decrease if the frequency of the health problem declines [16], [18]. Kassebaum et al. [3] speculate that massive declines in the global prevalence and incidence of total tooth loss between 1990 and 2010 are due to changes in societal and cultural norms as well as coordinated efforts in treating oral diseases and preventing tooth loss throughout life. More importantly, the situation of increasing relative but decreasing absolute inequality also occurs when the rate of improvement is smaller for the group with the worst initial health [35]. In our case, total tooth loss declined by 80% for the highest social class and 48% for the lowest social class. Taken together, our findings indicate that the significant decline in total tooth loss in the UK over the past two decades has not benefited all social classes evenly.

The magnitude of social inequalities in tooth loss is unlikely to change quickly [7]. Tooth loss reflects both the individuals’ history of dental diseases and treatment by dental services over the life course. As such, it captures a lifetime cumulative experience. To eliminate social inequalities in tooth loss, the determinants of inequalities must be identified and avoidable determinants modified. More attention should be directed at preventing dental diseases at all stages of the life course and at addressing the common determinants of chronic diseases, including oral diseases.

Tracking social inequalities in oral health over time is important to inform social and oral health-related policy. This is the first study to quantify and provide time comparative analysis of social inequalities in tooth loss using rigorous measures of social inequalities in oral health. As all previous trend analyses on social inequalities in tooth loss have relied on the range measure of inequality, their results are difficult to compare with ours. In line with previous recommendations [15], [17], [18], [36], future studies should report both absolute and relative measures of social inequalities in oral health.

Our findings should be interpreted in the light of some potential limitations. First, our findings were based on participants with complete data, which may raise concerns about representativeness of the data. However, the two analytical samples represented 93% and 94% of the adults and dentate adults that participated in the three surveys in England, Wales and Northern Ireland, respectively. In addition, the two samples were comparable with the general adult population in the three British countries in terms of demographic characteristics and social classes, which provides support for its representativeness and the generalization of the findings. Second, we used occupation-based social class to measure socioeconomic circumstances, ignoring other socioeconomic indicators relevant to oral health, such as education or income. A key strength of RGSC is its past official status in the UK, and hence, its widespread use in vital statistics and censuses over a long period of time. More importantly, it has been widely used to describe the socioeconomic gradient in health [37], [38]. Third, in 2001 NS-SEC replaced RGSC as the official socioeconomic classification in the UK, which may raise concerns about the validity of comparisons between the old and new socioeconomic classifications. However, Rose and Pevalin [30] constructed derivation matrices for RGSC from NS-SEC to assist with maintaining comparability across surveys and showed that 87 per cent of cases in NS-SEC can be allocated to the correct RGSC category. Further studies should explore whether similar trends of social inequalities in oral health are observed using alternative socioeconomic measures and oral health outcomes over longer periods of evaluation.

## Conclusions

There were pervasive inequalities in tooth loss by social class among British adults despite marked improvements in tooth retention in recent years and generations. In the whole population, absolute inequalities in total tooth loss have narrowed while relative inequalities have increased steadily. Among dentate adults, absolute and relative inequalities in number of teeth and proportion of people with functional dentition have remained significant, but unchanged over time.
